# Rotavirus antigenemia as a common event among children hospitalised for severe, acute gastroenteritis in Belém, northern Brazil

**DOI:** 10.1186/s12887-019-1535-2

**Published:** 2019-06-12

**Authors:** Maria Cleonice A. Justino, Erika A. Campos, Joana D’arc P. Mascarenhas, Luana S. Soares, Sylvia de Fátima S. Guerra, Ismari P. Furlaneto, Manoel Jaime C. Pavão Jr, Tassio S. Maciel, Fredison P. Farias, Orvácio M. Bezerra, Caio Breno G. Vinente, Rodrigo José S. Barros, Alexandre C. Linhares

**Affiliations:** 1 Instituto Evandro Chagas, Health Surveillance Secretariat, Brazilian Ministry of Health, Rodovia BR 316, Km 7, s/n, Levilândia, Belém, 67.030-000 Brazil; 2 Federal University of Pará State, Belém, Brazil

**Keywords:** Rotavirus, Gastroenteritis, Antigenemia, RNAemia, Hospitalisation

## Abstract

**Background:**

Rotavirus antigenemia and RNAemia (the presence of rotavirus RNA in serum) have been commonly identified among paediatric patients with acute gastroenteritis. In this study we examined the association between rotavirus antigenemia and clinical features, and sought to determine the genotypes of rotaviruses detected in paired stool and serum samples.

**Methods:**

Paired stool and serum samples were obtained from children hospitalised for acute gastroenteritis in Belém, Brazil, between June 2012 and June 2015. The 20-point Vesikari scoring system was used to assess the disease severity upon a retrospective medical record review. Stool and serum samples were primarily screened for the presence of rotavirus antigen using a commercial ELISA assay. The rotavirus isolates from stool and serum samples were genotyped by using the classical reverse-transcriptase polymerase chain reaction (RT-PCR) and/or through nucleotide sequencing of VP4 and VP7 genes. Viral load was estimated using real-time RT-PCR.

**Results:**

In total rotavirus antigen was detected in 109 (24.2%) stool samples from 451 children, whereas antigenemia occurred in 38.5% (42/109) of these patients. We demonstrated that patients positive for rotavirus RNA in paired stool and serum samples were more likely to have a higher frequency of vomiting episodes in a 24-h period (*p* = 0.0035). Our findings also suggested that children not vaccinated against rotavirus are more likely to develop antigenemia, as compared to those given at least one vaccine dose (*p* = 0.0151). G12P [8] and G2P [4] genotypes were predominant throughout the study period, accounting for 52.3% (57/109) and 27.5% (30/109) of the typed isolates, respectively. Ten stool-serum pairs could be typed for VP4 and VP7 genes. Seven of these pairs showed concordant results with G2P [4] genotype being detected in stool and serum samples, whereas discrepancies between genotypes (G2P [4]/G2P[NT] and G12P [8]/G2P[NT]) were seen in three pairs.

**Conclusions:**

Rotavirus antigenemia and RNAemia occur in a significant number of children hospitalised for acute gastroenteritis in Belém, Brazil, and may contribute to a greater disease severity, particularly translated into a greater number of vomiting episodes. This study documented a high concordance of genotypes detected in a subgroup of paired stool and serum samples.

**Electronic supplementary material:**

The online version of this article (10.1186/s12887-019-1535-2) contains supplementary material, which is available to authorized users.

## Background

Worldwide rotavirus still remains a major cause of deaths and hospitalisations due to gastroenteritis among children younger than five years, even though almost 100 countries have incorporated to date rotavirus vaccination into their national immunisation programs (NIPs) [1, 2]. According to recent estimates from the Global Burden of Disease Study, 2015 [3], around 260,000 deaths are attributable to rotavirus annually, most of which (~ 90%) occurring in the low-income countries of Asia and Africa.

Typically the clinical course of rotaviral enteritis can show a wide spectrum of symptoms ranging from mild, watery diarrhoea to severe gastroenteritis with vomiting, fever, abdominal pain and dehydration which may last 3 to 8 days [4, 5]. Although rotaviruses primarily infect mature enterocytes of the upper small intestine, there is increasing evidence pointing to their potential for spreading beyond the gastrointestinal tract, leading to the appearance of unusual extra-intestinal manifestations, namely febrile seizures and central nervous diseases such as encephalitis. [6–8].

It has been described that rotavirus RNA (which may imply viraemia) and antigen(s) (namely VP6) are detected in 64–93% and 33–90% in serum of children with rotavirus gastroenteritis, respectively [9–11]. Systemic spreading of rotavirus has been evidenced through the detection of antigen and/or RNA in multiple organs and tissues, possibly translating into the occurrence of a variety of medically relevant extra-intestinal diseases [12]. Although yet a controversial issue, some studies support the notion that viraemia and/or antigenemia may result in greater clinical severity, particularly with regards to the intensity/frequency of fever, convulsion and vomiting episodes [10, 13, 14]

As based on a binary classification system that focuses on the outer layer VP7 and VP4 proteins there are currently 28 G (of glycoprotein) and 39 P (protease-sensitive) genotypes, respectively. Of these, only 12 G and 15 P types are commonly associated with infections in humans, particularly those strains bearing G1P [8], G2P [4], G3P [8], G4P [8], G9P [8] and G12P [8] specificities, which altogether account for greater than 80% of circulating rotaviruses [4, 15, 16]. The issue of whether or not rotavirus genotypes may be a determinant of extra-intestinal spread remains to be fully elucidated. While some authors have reported that children infected with G1 type are more prone to develop antigenemia, as compared with other types [14, 17], there have been studies in Bangladesh, India and the United States showing that no significant difference was seen between infecting genotypes among children with or without antigenemia/ viraemia [6, 9, 12, 18].

In the current post-rotavirus vaccination scenario, where two live, attenuated, oral rotavirus vaccines [Rotarix (GlaxoSmithKline Biologicals, Rixensart, Belgium); and RotaTeq (Merck & Co. Inc. Kenilworth, NJ)] are licensed for use in over 100 countries [19], studies aiming at demonstrating the real world impact of vaccination in terms of morbidity and mortality are strongly recommended. To date, post-licensure evaluations of vaccination impact on rotavirus gastroenteritis-related morbidity and mortality have in general been conducted based on the detection of antigen in stools. It has however been demonstrated that concomitant attempts of detecting rotavirus antigenemia in post-licensure epidemiological studies are likely to enhance surveillance of rotavirus cases, besides providing a more accurate assessment of vaccine effectiveness [20].

## Aim and objectives

This study aimed primarily at determining if children hospitalised for acute gastroenteritis with rotavirus RNA and/or rotavirus antigen in stools and serum samples were more prone to develop severe gastroenteritis symptoms, as compared to those with RNA and/or rotavirus antigen detected in stools only. We also sought to assess whether or not rotavirus genotypes recovered from stool samples are homologous to those detected in sera.

## Methods

### Patients and specimens collection

This was a prospective, hospital-based study involving children with acute gastroenteritis attending public/private hospitals in Belém, Northern Brazil, between June 2012 and June 2015: from June 2012 to March 2013 at *Clínica Pediátrica do Pará* (which has closed as from March 2013 onwards); and from April 2013 to June 2015 at *Clínica Pio XII*). The recruitment of these children took place in these large paediatric hospitals, which then accounted for over 50% of all hospital admissions for gastroenteritis in the Belém area, as based on our previous local surveillance studies [21].

The study was approved by the Independent Ethics Committee of the Instituto Evandro Chagas, Health Surveillance Secretariat, Brazilian Ministry of Health, and was conducted in accordance with the principles of the Declaration of Helsinki, as well as in compliance with the Good Clinical Practice guidelines.

Children eligible to participate in the study were those aged at least 12 weeks and born after March 6, 2006, when rotavirus vaccination was introduced nationally in Brazil [22]. For surveillance purposes a case of acute gastroenteritis was defined as the passage of three or more looser-than-normal or watery stools within the 24 h before presentation, requiring at least one overnight stay and intravenous rehydration therapy. We did not consider eligible for recruitment both nosocomial gastroenteritis cases and children with gastroenteritis lasting ≥14 days at presentation. Information on demographics, medical/vaccination history, feeding practices and specific symptoms were obtained upon receipt of signed informed consent forms from parents/guardians. The 20-point scoring system, as proposed by Ruuska and Vesikary[23], was used to grade the severity of clinical symptoms (diarrhoea, fever and vomiting) upon a retrospective medical record review; gastroenteritis episodes reaching clinical scores of 1–10, ≥11 and ≥ 15 were classified as mild/moderate, severe and very severe, respectively.

Both serum and stool samples for rotavirus detection were collected from each eligible patient within the first 48 h of hospitalisation, stored thereafter in coolers and promptly transported - under controlled temperature within the range of 2-8 °C - to the Virology Section at Instituto Evandro Chagas, where these specimens were kept at -20 °C until processing. We excluded from analyses those patients (*n* = 105) from whom either blood or stool samples only were obtained. All stool samples were routinely screened for the presence of VP6 rotavirus antigen using a previously validated [24] sandwich-type commercial ELISA assay (RIDASCREEN R Rotavirus; R-Biopharma, Darmsdadt, Germany), according to manufacturer’s instructions. The same method was used to detect rotavirus antigen in undiluted sera, even though evidence of antigenemia was determined if the sample yielded an optical density, at a wavelength of 450 nm, equal to the mean OD of negative controls plus ≥2 standard deviations, in accordance with procedures described elsewhere [6, 17, 25].

Following viral genomic RNA extraction from ELISA-positive stool and serum specimens, amplification of the gene segments encoding for the VP4 and VP7 antigens was performed through a classical two-step reverse-transcription polymerase chain reaction (RT-PCR), using first round consensus primers 4Con3-4Con2 and Beg9-End9, respectively, as described elsewhere [26, 27]. Stool samples were subjected to a second, nested-RT-PCR step including well-established oligonucleotide primers targeted at G (G1, G2, G3, G4, G9 and G12) and P (P [4], P [6], P [8], and P [9]) rotavirus genotypes [26, 28, 29]. In addition, a second, nested-PCR step was performed with sera using VP7F and VP7R primers, according to the methods described earlier [30]. The resulting bands could be visualised following gel electrophoresis and staining with SYBR Safe™ DNA gel stain. In order to measure the rotavirus load in stool and serum samples we performed a reverse-transcriptase quantitative PCR (qRT-PCR), using the primers and TaqMan probe targeting a highly conserved region of the non-structural protein 3 (NSP3) of rotavirus, essentially as described before [31]. The reaction was performed on an ABI 7500 genetic analyser (Applied Biosystems, Foster City, CA).

To determine the electropherotypes the extracted dsRNAs from stool samples were also further electrophoresed in a 5% polyacrylamide gel, followed by silver staining as previously described [32].

Nucleotide sequencing was carried out with rotavirus-positive serum samples that could not be genotyped previously using conventional RT-PCR. Eleven pairs of stool and serum specimens were subjected to nucleotide sequencing for VP7 and VP4 genes with a Big Dye Terminator cycle sequencing kit v 3.1 (Applied Biosystems, Foster City, CA). Electrophoresis was performed in the ABI Prism 3130xl automatic sequencer (Applied Biosystems) and the sequences obtained were aligned and edited using the BioEdit Sequence Alignment Editor program (v. 7.0.5.2). Neighbour-joining method was used to perform the phylogenetic analysis, in which distance was calculated from aligned sequences. The nucleotide sequences reported in this study have been deposited in the GenBank sequence database and assigned the following accession numbers: MH456983 to MH456986.

Statistical analyses were carried out using the BioEstat v5.0 software package [33]. Comparisons of individual laboratory results, clinical symptoms and severity scores between patients with and without rotavirus antigenemia were performed using χ^2^ tests or G test when appropriate for categorical data. The Student’s t-test or the Mann-Whitney U test was used for comparison of two independent sets of continuous, quantitative data. The Spearman’s rank correlation coefficients (rs) were used to measure the strength of the relationship between either antigenemia or RNAemia concentrations and clinical and laboratory parameters. The Spearman’s rank correlation was also used to assess the relationship between viral load in stool samples and levels of antigen in sera. We considered *P* values of less than 0.05 as being statistically significant.

## Results

**Fig. 1:**
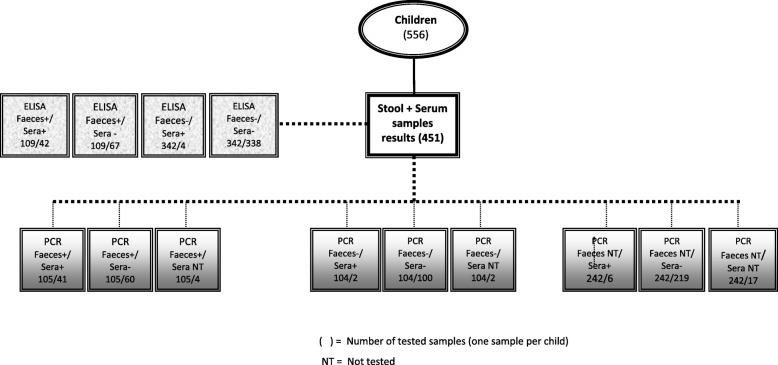
Study profile summarising laboratory tests (ELISA and RT-PCR) performed with faeces and/or serum samples from 451 (of 556) children in Belém, Brazil

During June 2012 through June 2015, we identified 3740 children who were age-eligible to potentially participate in the study. Of these, a total of 556 paediatric inpatients with acute gastroenteritis were enrolled to participate in this study. Paired serum and stool samples could be obtained from 451 subjects. Paired samples from the former subgroup were tested by ELISA and qRT-PCR (quantitative RT-PCR) as demonstrated in Fig. 1. By ELISA, 109 (24.2%) stool samples out of the 451 subjects were rotavirus-positive; and among these rotavirus-positive children 38.5% (42/109) had detectable rotavirus antigen in their sera (antigenemia). Conversely, antigenemia was detected in four patients with rotavirus-negative stool samples. The analysis of stool samples by qRT-PCR yielded rotavirus RNA in 105 of patients and also in serum among 41 of these children, representing 39% of RNAemia in this subgroup. Stool samples from 242 patients could not be tested (NT) by qRT-PCR In addition, RNAemia was detected in 8 patients among those with stool samples either negative or not tested by PCR.

In order to explore potential indicators of antigenemia, we compared the clinical characteristics and the vaccination status between rotavirus-related acute gastroenteritis patients with rotavirus-positive (*n* = 42) and rotavirus-negative (*n* = 67) sera, by ELISA (Table 1). As based on the Vesikari scoring system, a trend for greater clinical severity was seen among children with rotavirus-positive serum, as compared to those with rotavirus-negative serum (*p* = 0.1557). With regards to specific clinical parameters, the only statistically significant difference was observed for the number of vomiting episodes in a 24-h period: patients positive for rotavirus RNA in paired stool and sera were more likely to have ≥3 vomiting episodes/24 h (*p* = 0.0035). Our findings also suggest that children not vaccinated against rotavirus were more likely to have antigenemia than those given at least one vaccine dose (*P* = 0.0151). Extra-intestinal involvement of rotavirus infection was not seen in this study.

**Table 1:** Comparison of clinical characteristics and vaccination status between rotavirus-related acute gastroenteritis cases with and without rotavirus antigen in serum samples

Characteristics [N*]	Children with rotavirus-positive stools (N = 109)				P value**
	Rotavirus-positive in paired sera		Rotavirus-negative in paired sera		
	N (%)	95% CI	N (%)	95% CI	
Ruuska & Vesikari score^†‡^ [105]					
≥11 (severe)	13 (30.9)	19.1-46.0	29 (46.0)	34.3-58.2	0.1557
≥15 (very severe)	29 (69.1)	53.9-80.9	34 (54.0)	41.8-65.7	
Axillary temperature (°C)^£^ [107]					
<37.5	11 (26.2)	15.3-41.1	18 (27.7)	18.3-39.6	1.0000
≥37.5	31 (73.8)	15.3-41.1	47 (72.3)	60.4-81.7	
Episodes of vomiting/24h [109]					
≤3	7 (16.7)	8.3-30.6	30 (44.8)	33.5-56.6	0.0035
>3	35 (83.3)	69.4-91.7	37 (55.2)	44.4-66.5	
Duration of vomiting (days) [109]					
≤3	34 (80.9)	66.7-90.0	55 (82.1)	71.3-89.5	1.0000
>3	08 (19.1)	9.9-33.3	12 (17.9)	10.6-28.8	
Episodes of diarrhoea/24h^§^ [108]					
≤3	5 (11.9)	5.2-25.0	13 (19.7)	11.9-30.8	0.4277
>3	37 (88.1)	75.0-94.8	53 (80.3)	69.2-88.1	
Duration of diarrhoea (days) [109]					
≤3	31 (73.8)	58.9-84.7	47 (70.0)	58.3-79.8	0.8278
>3	11 (26.2)	15.3-41.1	20 (30.0)	20.2-41.7	
Leucocytes/ mm^3||^ [108]					
≤10.000	31 (73.8)	58.9-84.7	56 (84.8)	74.3-91.6	0.2126
>10.000	11 (26.2)	15.3-41.1	10 (15.2)	8.4-25.7	
Rotavirus vaccination^¶^ [85]					
Unvaccinated	8 (25.8)	13.7-43.3	3 (5.6)	1.9-15.1	0.0151
At least one dose	23 (74.2)	56.8-86.3	51 (94.4)	84.9-98.1	

*Number of patients with available data for evaluation

**Fisher test

^**†**^20 points Ruuska & Vesikari score (1990)

^**‡**^ Not available for 2 patients; moderate score for 2 patients, not included

**£** Not available for 2 patients

^**§**^ Not available for 1 patient

^**||**^ Not available for 1 patient

^**¶**^ Vaccination status not available for 24 patients

**Fig. 2:**
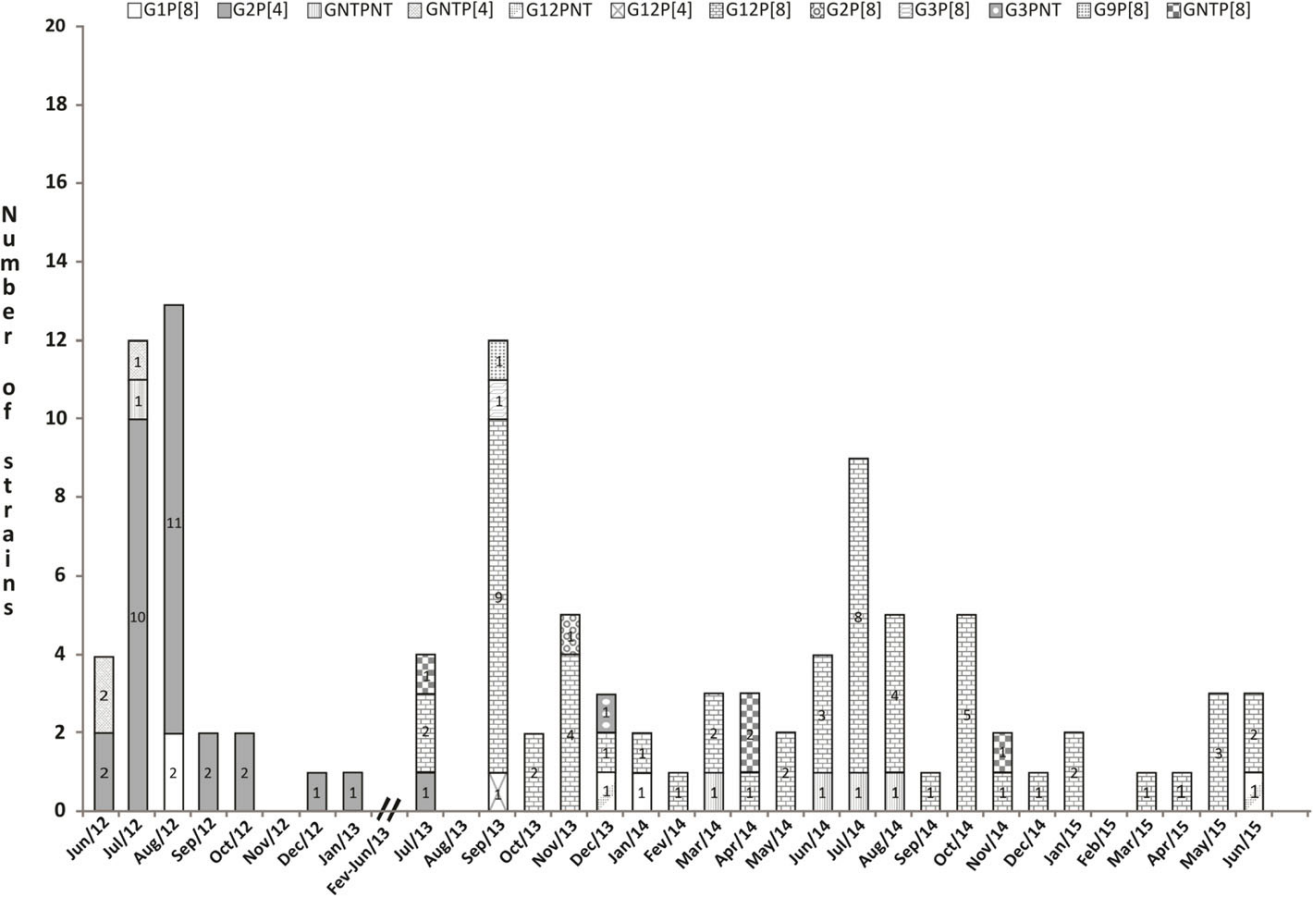
Annual distribution of rotavirus genotypes identified in faecal samples from 109 children with gastroenteritis in Belém, Brazil

Genotyping was done on the 109 rotavirus-positive stool samples to determine G and P types, as shown in Fig. 2. Altogether G12P [8] and G2P [4] rotavirus strains were largely predominant throughout the study period, accounting for 52.3% (57/109) and 27.5% (30/109) of the isolates, respectively. The circulation of G2P [4] strains was observed from June 2012 through July 2013, with peak incidence rates in July (83.3% of genotyped strains) and August 2012 (84.6%), decreasing sharply thereafter until July 2013, in line with the overall rotavirus positivity rates. Rotavirus strains with G12P [8] genotype specificities emerged in July 2013, with rates increasing significantly in the following months and reaching incidence rates as high as 75% in September 2013 and 88.9% in July 2014. A variety of other rotavirus genotypes co-circulated at very low frequencies, concurrently with the predominant G2P [4] and G12P [8] strains, particularly during July 2013 through June 2015. G1P [8] rotavirus strains accounted for an overall 2.7% prevalence rate but were found to circulate in August 2012 and January 2014. The remainder of the circulating strains belonged to G12P [4], G2P [8], G3P [8], and G9P [8] genotypes and accounted for < 4% of all typed isolates. Partially typed or fully untypeable rotavirus strains were found during July 2013–June 2015 only, altogether accounting for around 10% of all isolates.

**Fig. 3:**
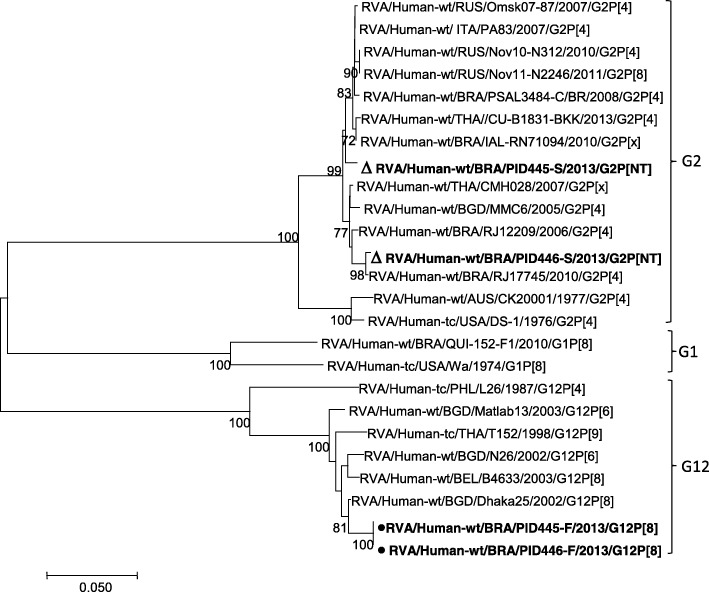
Phylogenetic dendogram of partial VP7 gene of G2P [4] and G12P [8] strains from stool and serum samples (highlighted in bold) during June 2012–June 2015 in Belém, Brazil. Bootstrap values (2000 replicates) are shown at the branch nodes; values < 70% are not shown. Strains detected in serum samples are indicated with empty triangles while strains detected in stool samples are indicated with black circles

Table 2 shows a comparison between G- and- P- type-specificities in paired stool-serum samples from ten individual patients admitted for rotavirus-related gastroenteritis. There were fully concordant results in seven of the eight stool-serum pairs in which rotavirus G2P [4] genotype was identified. Among these, a potential discrepancy was seen in one pair with identification of G2P [4] and G2P[NT] genotypes in stool and serum samples, respectively. Rotavirus strains with discordant G-type and P-type specificities were detected in two pairs: G12P [8] and G2P[NT] genotypes in stools and sera, respectively. Among the G2 concordant pairs all electropherotypes displayed identical short patterns, while a long profile was associated with the two discordant G12P [8] and G2P[NT] pairs. In order to confirm the latter discrepancy in the stool-serum pairs, as determined by RT-PCR, a sequence analysis of the VP7 gene was performed on these two samples (Fig. 3).

**Table 2:** Distribution of rotavirus genotypes in paired stool and serum samples obtained from 10 individual patients hospitalised for gastroenteritis in Belém, Brazil

Patient identification	Electropherotypes in stool samples	Rotavirus G and P genotypes	
		Stool samples	Serum samples
PID 34	Short	G2P[4]	G2P[4]
PID 37	Short	G2P[4]	G2P[4]
PID 39	Short	G2P[4]	G2P[4]
PID 40	Short	G2P[4]	G2P[4]
PID 79	Short	G2P[4]	G2P[4]
PID 87	Short	G2P[4]	G2P[4]
PID 93	Short	G2P[4]	G2P[4]
PID 353	Short	G2P[4]	G2P[NT]
PID 445	Long	G12P[8]	G2P[NT]
PID 446	Long	G12P[8]	G2P[NT]

*NT* Not typed

Phylogenetic analysis of the partial VP7 gene of G2 sequences from serum samples of subjects PID 445 and PID 446 showed high sequence identities (97.8–100%) with strains of human origin from Brazil, Russia, Bangladesh, Italy and Thailand. In addition, G12 genotypes detected concurrently in stool samples from PID 445 and PID 446 patients were shown to be closely related (95.6–98.5%) to human strains from Bangladesh, Thailand and Belgium. Serum samples from ten diarrhoeic children with rotavirus-positive stools yielded clear strong amplicons of 881 base pairs (bp) and 876 bp, of the genes encoding VP7 and VP4 proteins, respectively, as depicted in Additional file 1: Figure S1.

**Fig. 4:**
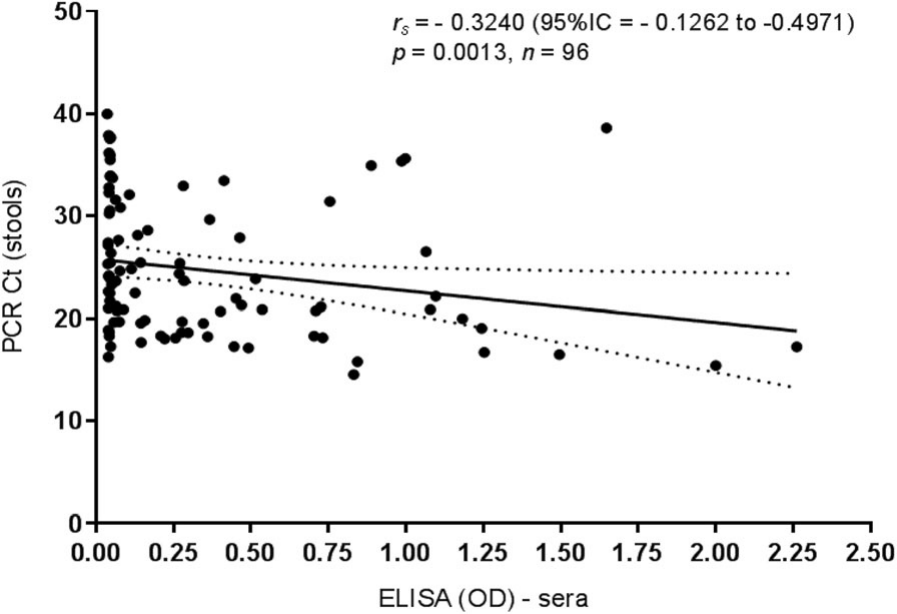
Correlation between stool viral load as determined by PCR cycle Ct values, and the rotavirus antigen levels in serum samples from 96 children with gastroenteritis in Belém, Brazil, as assayed by ELISA

As demonstrated in Fig. 4, there was a moderate negative correlation between the stool viral load as determined by qRT- PCR cycle Ct values, and the rotavirus antigen levels expressed as optical densities (OD) in sera tested by ELISA [*n* = 96 paired samples; Pearson’s correlation coefficient (rs) = − 0.3240; 95% CI − 0.1262 to − 0.4971; and *p* value = 0.0013].

## Discussion

In spite of the current scenario where two licensed vaccines are increasingly being made available for introduction in many countries around the world, there remains to be fully elucidated the mechanisms of pathogenesis in rotavirus infection, particularly that underlying virus spreading beyond the gastrointestinal tract into the blood stream [6]. The current study’s main focus lies on the potential impact of rotavirus antigenemia on clinical manifestations in a cohort of children hospitalised for acute gastroenteritis in Belém, Brazil. Since current available evidence points to the fact that antigenemia and RNAemia are common events among children infected with rotavirus, we chose to use ELISA (targeted mainly at the VP6 protein) and qRT-PCR/RT-PCR in order to determine the presence and characterise rotavirus strains in both faecal and serum samples.

Noticeably in our cohort, rotavirus antigen was detected in 24% of the stool specimens tested by ELISA, an overall positivity rate found to be higher than that of 17% reported in a study also conducted during the early years post-rotavirus vaccine introduction in Brazil [34]. The distinct field/laboratory methodologies used in these studies, however, may have accounted for such difference in prevalence rates. A finding that deserves further consideration is the fact that one fifth of the children in our study cohort developed severe rotavirus-related gastroenteritis, despite a rotavirus vaccination coverage rate of around 90% in our setting. Since previous studies in our region have suggested that children who undergo a full vaccination schema are less likely to develop rotavirus infection requiring hospitalisation [35], an incomplete immunisation schedule could possibly have accounted for such unexpected high rate of severe rotavirus-related gastroenteritis. Nonetheless, we cannot rule out in this context the potential challenge represented by the globally emerging G12P [8] genotype, which was found to be the dominant circulating rotavirus strain during a two-year surveillance period (June 2013–July 2015) in our study.

Our results showed that antigenemia was present in around 40% of children hospitalised for acute RV-related gastroenteritis in Belém, a rate similar to those reported in previous studies conducted elsewhere which were in the range of 33–75% [10, 13, 17, 18, 36]. Our data showed that 40% of patients with a rotavirus-positive stool sample were found to have rotavirus RNA in serum, a rate lower than those rates from studies conducted elsewhere which also included either blood samples or plasma and – unlike our study – were conducted before universal introduction of rotavirus vaccine [14]. Interestingly, our study suggests that ELISA and RT-PCR had similar sensitivities for the detection of antigenemia (positivity rate, 9.3%) and RNAemia (11.4%), respectively. These results appear to be in line with those from studies in India where comparable – even though higher – rates of antigenemia (71.2%) and RNAemia (81.9%) were detected among children with rotavirus-positive stool samples [37]. Unlike previous studies [36] which have recently reported a higher prevalence rate of RNAemia (70.8%), as compared to antigenemia (33.3%), in peripheral blood mononuclear cells (PBMC) and plasma, respectively, of children with acute diarrhoea in USA. Moreover, these authors utilised a different RT-PCR system where primers targeted specifically the rotavirus NSP4 gene.

As also reported elsewhere [17], we have demonstrated that rotavirus RNA levels detected in serum samples (11.4%) were significantly lower than RNA levels found in stool samples (50.7%), supporting the general notion that rotaviruses replicate primarily in the mature, differentiated enterocyte of the small intestine [38].

In our study we performed a correlation analysis of paired stool and serum samples obtained from 96 paediatric patients which provided evidence that the stool rotavirus copy numbers correlate significantly with the ELISA OD values in sera (Fig. 4). These findings are in line with those from Ramani et al. [8, 18] who also stated a positive correlation between the levels of rotavirus antigen in sera with the stool viral load. These authors have hypothesised that a higher stool viral load (and severe disease) would account for a major damage of the intestinal epithelium, resulting in the spread of rotavirus beyond the intestines into the circulatory system.

In the present study we were able to assess whether or not VP7 and VP4 genotype specificities were concordant in comparing paired stool-serum samples from ten paediatric patients hospitalised for rotavirus-related gastroenteritis during the first year of surveillance. Rotavirus strains bearing G2 and P [4] genotype specificities – which were found to be dominant during June 2012–July 2013 – could be identified in seven of the ten pairs, suggesting that rotaviruses infecting the gut were the same strains which disseminated beyond the intestine. These concordant results appear to be consistent with findings from a few other studies conducted elsewhere, even though discrepancy of genotypes in stool and serum samples (G12P [8] and G2P[NT] pairs) of individual patients was also see in our two of our study patients. These latter findings raise the hypothesis that identification of distinct genotypes in two specimens from a single patient may reflect either consecutive infections at different time points or the occurrence of intrinsic difference in growth characteristics and/or tissue tropism, leading to speculate that during co-infections a preferential extra-intestinal spread of some strains may occur [25, 39, 40]. In this context, for example, Hemming et al. [14] have suggested that both antigenemia and RNAemia are particularly common in severe rotavirus gastroenteritis caused by G1P [8] genotype. Nonetheless, the issue of whether or not the potential for rotavirus antigenemia/viraemia is limited to specific genotype(s) remains largely controversial [41]. In our study, the discrepancy among genotypes present in different specimens from the same individual was clearly confirmed through sequence analysis of the VP7 gene of the two samples, as also reported elsewhere [25]. Additionally in support to the discordant genotypes was the identification of short and long electrophoretic profiles for the G2 and G12 strains present in the same specimen, respectively.

In the present study we also sought to determine whether children with antigenemia presented with more severe rotavirus disease than children without antigenemia. It was observed that patients with rotavirus antigen in serum were more likely to develop frequent vomiting, as compared to patients without antigenemia. In contrast, we could not find any significant difference between the groups in regards to the severity of other clinical parameters including fever, duration of vomiting, diarrhoea and white blood cell count. Unlike other studies conducted elsewhere [8, 18], our findings did not show any significant difference in the overall disease severity, as graded by the Vesikari clinical scoring system, between children with antigenemia and children without antigenemia. Of note, our observation that rotavirus antigenemia may specifically correlate with increased severity of vomiting is consistent with findings from recent studies in Finland and India [14, 37]. Since the rotavirus-induced emesis involves secretion of serotonin from enterochromaffin cells with stimulation of vagal afferent nerves connected with the brain stem, it has been speculated that antigenemia/RNAemia might reflect an infection deeper into the gut, possibly leading to a more powerful activation of the brain areas essential for vomiting [14, 42, 43].

Of potential importance in the current post-rotavirus vaccine introduction scenario, our findings suggest that children not vaccinated against rotavirus appear to be more likely to develop antigenemia, as compared to those given at least one vaccine dose. However, further studies are needed to confirm this finding since such a difference may have occurred by chance owing to the small sample size in our study.

Although a significant proportion of children in our study were found to develop antigenemia/RNAemia, we could not detect any clinical manifestations which might be suggestive of extra-intestinal involvement of rotavirus infection. This supports the notion that while viraemia constitutes a common event, it does not appear that systemic infection is frequently associated with clinically significant non-gastrointestinal disease. Furthermore, it has also been postulated that subclinical extra-intestinal infections often occur in children and may occasionally progress to an overt clinical condition [6, 12, 44, 45].

The findings of this study are subject to some limitations. First, our antigenemia results should be interpreted with caution, since all currently available ELISA commercial kits are designed to detect rotavirus antigen in stool samples and had not been validated for use with either human or plasma samples. Nonetheless, our data suggest that ELISA and RT-PCR display similar sensitivities for the detection of antigenemia and RNAemia, respectively. Of note in this regard, earlier studies have suggested that ELISA may potentially offer a new practical diagnostic tool for the detection of rotavirus from serum/plasma samples in situations where a stool sample is not readily available (e.g. severe dehydration) [13]. Second, we were unable to demonstrate the presence of infectious virus particles in the serum samples using cell cultures for rotavirus isolation, which might reflect a true viremia. It should be pointed out however that RNA detection in blood has been reported as a strong indicator of the presence of viraemia among children with rotavirus gastroenteritis [6]. In addition, the current study did not include the search for differences at both nucleotide and amino acid levels of the paired strains under comparison, an aspect which is worth considering further.

In summary, our results confirm and extend previous well documented findings that rotavirus antigenemia/ RNAemia occurs routinely among children with acute rotavirus gastroenteritis and may contribute to a greater disease severity by increasing the number of vomiting episodes. Of particular interest in the current context of growing rotavirus vaccine introduction, it is has been pointed out that detection of antigenemia/RNAemia may represent an additional, useful approach to improve the sensitivity of post-licensure epidemiological studies of vaccine effectiveness [20]. In addition, the detection of rotavirus antigenemia/RNAemia seems also warranted as it remains to be determined whether any currently used rotavirus vaccine strains may eventually spread beyond the intestine.

## Conclusions

To our knowledge this is the first report in Brazil showing that rotavirus antigenemia and RNAemia are detectable in a significant proportion of infants and young children admitted to hospital due to acute gastroenteritis. According to the Vesikari scoring system a trend for greater clinical severity was seen among children with rotavirus-positive serum, as compared to those with rotavirus-negative serum. A statistically significant difference between these groups was particularly seen with regards to the number of vomiting episodes. It has also been demonstrated a positive correlation between the levels of rotavirus antigen in sera, as determined by ELISA, with the stool viral load, as assessed by qRT-PCR. Both G2P [4] and G12P [8] rotavirus genotypes accounted for most of the isolates throughout the study period; in addition, we have documented a high concordance of genotypes detected in a subgroup of paired stool and serum samples. Further studies on rotavirus antigenemia/RNAemia are warranted to better assess the effectiveness of currently used rotavirus vaccines, as well as to determine whether licensed vaccines may eventually spread beyond the intestines.

## Additional file

Additional file 1: **Figure S1.** Agarose gel electrophoresis of RT-PCR VP7 (A) and VP4 (B) gene products obtained from the sera of ten rotavirus-positive children with gastroenteritis in Belém, Brazil. (PPTX 121 kb)

## Data Availability

Raw data of the study are available from the corresponding author on reasonable request. For this, a permission from MCAJ would be needed. The nucleotide sequences of VP7 genome segments of two representative G2 strains and two representative G12 strains collected in this study in Belém, Brazil, are available in the GenBank, through accession numbers MH456983 to MH456986.
