# Supplementary figures and images for: Rotavirus antigenemia as a common event among children hospitalised for severe, acute gastroenteritis in Belém, northern Brazil
Source: BMC Pediatr. 2019 Jun 12;19:193. doi: 10.1186/s12887-019-1535-2 (PMC6560848; doi:10.1186/s12887-019-1535-2)

## Slide 1
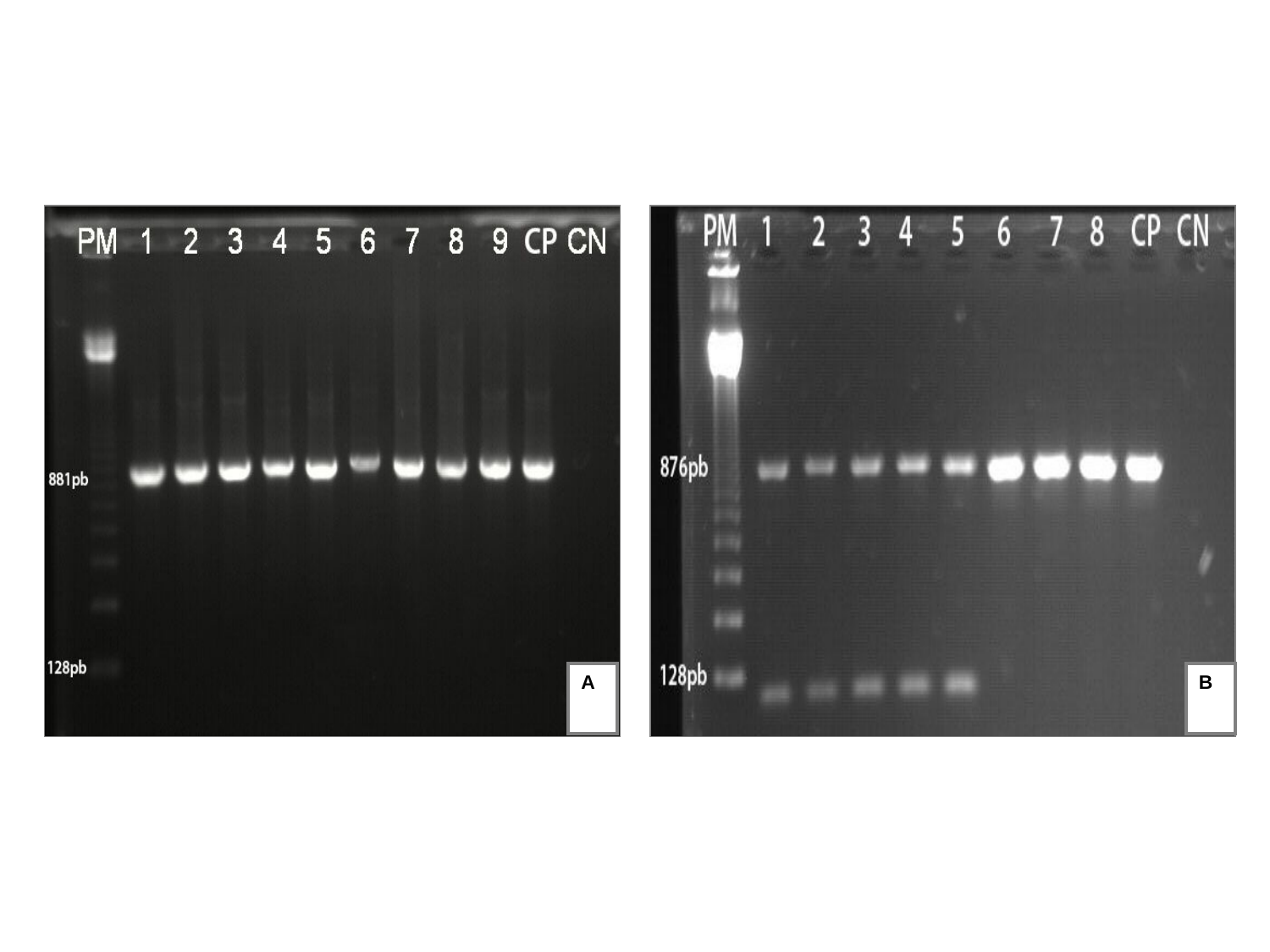

A
B

Supplement: Supplementary file 1 — Figure S1. Agarose gel electrophoresis of RT-PCR VP7 (A) and VP4 (B) gene products obtained from the sera of ten rotavirus-positive children with gastroenteritis in Belém, Brazil. (PPTX 121 kb) [file 12887_2019_1535_MOESM1_ESM.pptx]
